# Women's Childbirth Experiences in the WILL Randomised Trial (When to Induce Labour to Limit Risk in Pregnancy Hypertension): A Mixed Methods Analysis

**DOI:** 10.1111/1471-0528.18257

**Published:** 2025-06-24

**Authors:** Sue Tohill, Katie Kirkham, Eleni Gkini, Catherine A. Moakes, Sergio A. Silverio, Gillian Horgan, Ben Wills, Jennifer A. Hutcheon, Joel Singer, Clive Stubbs, Jim G. Thornton, Peter von Dadelszen, Laura A. Magee, the WILL Trial Study Group

**Affiliations:** ^1^ Maternity Services, Guy's and St Thomas' National Health Service (NHS) Foundation Trust London UK; ^2^ Birmingham Clinical Trials Unit University of Birmingham Birmingham UK; ^3^ Department of Psychology, Institute of Population Health University of Liverpool Liverpool UK; ^4^ Department of Women & Children's Health, School of Life Course & Population Sciences King's College London London UK; ^5^ Sands Charity London UK; ^6^ Department of Obstetrics and Gynaecology University of British Columbia Vancouver British Columbia Canada; ^7^ School of Population and Public Health and Centre for Health Evaluation and Outcome Sciences University of British Columbia Vancouver British Columbia Canada; ^8^ Department of Obstetrics and Gynaecology University of Nottingham Nottingham UK

**Keywords:** childbirth experience, chronic hypertension, gestational hypertension, hypertension in pregnancy, labour induction, maternal complications, neonatal morbidity

## Abstract

**Objective:**

To compare childbirth satisfaction in women with chronic or gestational hypertension, randomised to ‘planned early term birth at 38^+0–3^ weeks' gestation’ (intervention) or ‘usual care at term’ (control).

**Design:**

Randomised trial.

**Setting:**

Forty‐two consultant‐led maternity units, United Kingdom.

**Population:**

357/403 women randomised completed the Childbirth Experience Questionnaire (CEQ).

**Methods:**

Mixed‐methods analysis of the 22‐item CEQ, assessing: ‘Own capacity’, ‘Professional support’, ‘Perceived safety’ and ‘Participation’. Directed content analysis sorted free‐text comments into themes covered by the CEQ and two additional themes.

**Main Outcome Measures:**

CEQ scores overall and by domain.

**Results:**

In intervention (vs. control) groups, the CEQ was completed by 177/202, 88.1% (vs. 180/202, 89.1%) participants, and 378 free‐text comments were made by 93/177, 52.5% (vs. 98/180, 54.4%) participants. There was no significant difference in CEQ scores overall (3.1 ± 0.4 vs. 3.1 ± 0.4, respectively) or by domain (‘Own capacity’ [2.8 ± 0.5 vs. 2.7 ± 0.5, respectively]; ‘Professional support’ [3.7 ± 0.5 vs. 3.7 ± 0.6, respectively]; ‘Perceived safety’ [3.2 ± 0.6 vs. 3.1 ± 0.6, respectively]; and ‘Participation’ [2.6 ± 0.7 vs. 2.7 ± 0.6]). Most comments were positive (222/378, 58.7%), and about ‘Relational care and care interactions’ (CEQ ‘Professional support’). Neither the number nor positivity of comments appeared to differ between groups.

**Conclusion:**

For women with chronic or gestational hypertension who remain well at term, we found no difference in childbirth experience between women randomised to planned early term birth versus usual care at term. Shared decisions about timing of birth may be more influenced by differences in clinical outcomes and costs.

**Trial Registration:**

ISRCTN: 77258279

## 1 Introduction

Chronic or gestational hypertension complicates ≈ 7% of pregnancies [1], half of which will reach 37 weeks' gestation [2]. Observational data suggest that early term birth (at 37 to 38 weeks) may reduce maternal complications (e.g., pre‐eclampsia), Caesareans, stillbirths [3, 4, 5, 6] and costs of maternal‐fetal surveillance [7]; however, early term birth may increase neonatal morbidity [8]. There are no high‐quality data on which to base timing of birth for this high‐risk population.

The WILL trial (When to Induce Labour to Limit risk in pregnancy hypertension) aimed to address the optimal timing of birth for women with chronic or gestational hypertension at term gestational age, when women remain well and there is no evidence of pre‐eclampsia [9]. WILL was a multicentre randomised trial of 403 women with chronic or gestational hypertension, who were randomised at 37^+0–6^ weeks' gestational age to either ‘planned early term birth at 38^+0–3^ weeks’ gestation' (*N* = 201, intervention group) or ‘usual care at term’ (*N* = 202, control group); the trial was stopped early by the funder due to slower‐than‐anticipated recruitment during the COVID‐19 pandemic. The clinical outcomes and costs favoured the intervention group [9]. While in the intervention (vs. control) group, there was no difference in the co‐primary outcomes of ‘poor maternal outcome’ (severe hypertension, maternal death, or maternal morbidity; 27, 13% vs. 24, 12%, respectively) or ‘neonatal unit admission for ≥ 4 h’ (14, 7% vs. 14, 7%, respectively), or Caesarean births (58, 29% vs. 72, 36%, respectively), there was a significant reduction in pre‐eclampsia (56, 27.9% vs. 76, 37.6%, respectively) and costs for tests of maternal or fetal wellbeing (−£102.84, 95% CI −136.65 to −67.78).

In addition to clinical outcomes and costs, patients' experiences were evaluated in the WILL trial [9]. As with all health policy, it is important that timing of birth recommendations be associated with positive psychosocial outcomes for women, particularly as dissatisfaction with the childbirth experience has been associated with negative consequences, such as on breastfeeding, infant bonding and postpartum mental health [10].

In this article, we describe the experiences of women in the WILL trial, as evaluated by the Childbirth Experience Questionnaire (CEQ) and their associated free‐text comments.

## 2 Methods

This paper reports on the childbirth experiences of participants within the WILL trial who were recruited from June 2019 to December 2022, from 44 National Health Service (NHS) maternity units in the United Kingdom, where care was overseen by senior obstetricians. The WILL trial protocol [11] and results [9] have been published previously and described above (ISRCTN 77258279).

This report focuses on maternal satisfaction with care, evaluated by the CEQ [12]. WILL participants were asked to complete the CEQ prior to primary hospital discharge after birth; if this were not possible, research teams not involved in women's care were asked to mail the questionnaire to women at home, or assist them in completing the questionnaire over the phone. The CEQ was provided only in English, but if needed, women could ask family to support them with translation. The method of questionnaire completion was not documented.

### 2.1 Childbirth Experience Questionnaire

The CEQ was developed by Dencker et al. [12], to study women's perceptions of their first labour and birth and was validated for use in the United Kingdom by Walker et al. [13], to identify negative childbirth experiences. The CEQ is a 22‐item self‐administered questionnaire assessing four domains of childbirth experience: ‘Own capacity’ (8 items, which reflect the woman's sense of control, confidence and ability to cope during labour and birth), ‘Professional support’ (5 items, which assess the perception of support and encouragement received from healthcare providers, including midwives and obstetricians), ‘Perceived safety’ (6 items, which measure feelings of security and trust in the healthcare system and birth environment) and ‘Participation’ (3 items, which evaluate the extent to which the woman felt involved in decision‐making and had an active role in her care) (Table S2).

CEQ responses are assessed overall, as well as by domain, with 19 individual questions and statements (items) scored using a 4‐point Likert scale ranging from 1 (Totally agree), 2 (Mostly agree), 3 (Mostly disagree) to 4 (Totally disagree). Ratings of positively‐worded statements and the pain items are reversed so that higher scores reflect more positive scoring. Three items (i.e., questions 20–22) are assessed using a visual analogue scale from 0 to 10; these were presented as an integer scale, and coded as per published guidance, as 1 for scores of 0–4, 2 for scores of 5–6, 3 for scores of 7–8 and 4 for scores of 9–10.

The score of each of the four domains is calculated as the mean of the values of items. As some participants may not have a partner, one question had ‘not applicable’ as an option (with the permission of the author); for scoring, this is interpreted as missing. Overall scores range from 1 to 4, with higher scores representing a more positive childbirth experience. As the CEQ has not been validated for use in women giving birth by elective Caesarean, the participation domain score was not calculated for these women, whose overall mean score was the average of the other three domain scores. If at least half of the questions within a domain were answered, the half‐scale method was used whereby the sum of the scores was divided by the number of answered items; the overall CEQ score was then the mean of the four individual domain scores.

For overall and domain scores, means and standard deviations were reported alongside adjusted mean differences (with 95% CIs), estimated using a linear regression model, adjusted for the trial minimisation variables (of centre, hypertension type and prior Caesarean). For the primary statistical analysis, all women were included.

In a sensitivity analysis, the total and the subscale scores were presented for women who did and who did not have an adverse outcome, for the co‐primary outcomes (maternal and neonatal), key secondary outcome (Caesarean, overall and elective or non‐elective) and pre‐eclampsia (which occurred less commonly in the intervention [vs. control] group).

### 2.2 Free‐Text Comments

At the end of the CEQ, women were asked: ‘Please share with us any additional comments that you would like to make’, and were provided with space to add free‐text comments. These were analysed using a directed content analysis to further validate extant concepts, frameworks, or theories [14]. Data were analysed inductively, but within the parameters of further understanding the childbirth experience as measured by the CEQ, according to the four domains.

Directed qualitative content analysis was undertaken by the WILL research midwife (ST) who was not involved in the care of trial participants. Responses were sorted into clusters of ‘positive’, ‘negative’, or ‘both positive and negative’. Comments which were ‘entirely factual’ or ‘neutral’ (e.g., ‘*No further comments to add*’) were not included further in analyses. All data were coded to achieve 100% analytic coverage, with any additional themes beyond those of the CEQ added. Coding clusters were then broken down into more nuanced themes and sub‐themes, undertaken independently by KK. Differences in coding were discussed between ST and KK, and themes were developed, reviewed and agreed by study team members (LAM, KK, GH and SAS).

### 2.3 Patient and Public Involvement

The trial had two PPIE members in the Co‐Investigator Group (Table S1), a PPIE representative on the Trial Steering Committee, and a bespoke PPIE group (Acknowledgements) which reviewed patient and public‐facing material for trial promotion and recruitment.

## 3 Results

The results of the trial have been reported [9]. In brief, the planned sample size was 1080 women, but recruitment was delayed during the COVID pandemic, and as part of ‘post‐pandemic reset’, the funder directed the trial to stop, without knowledge of results. 403 participants had been randomised, 201 to planned early term birth (intervention) and 202 to usual care at term (control).

Most women underwent initiation of birth by labour induction, in intervention (87.1%) and control (68.8%) groups; timed birth was the intervention, usually delivered by labour induction, whereas in the control group, women were induced most commonly for maternal (39.5%) or fetal indications (21.7%). In the intervention (vs. control) group, there was no difference in the co‐primary outcomes of ‘poor maternal outcome’ (13.4% vs. 11.9%, respectively; adjusted risk ratio [aRR] 1.16, 95% CI [CI] 0.72–1.87) and ‘neonatal care unit admission for ≥ 4 h’ (7.0% vs. 6.9%, respectively; aRR 1.04, 95% CI 0.52–2.08), or the key secondary outcome of Caesarean birth (28.9% vs. 35.6%, respectively; aRR 0.81, 95% CI 0.61–1.08), but pre‐eclampsia was less common in the intervention (27.9%) vs. control group (37.6%) (aRR 0.74, 95% CI 0.56–0.98). Healthcare utilisation was lower in the intervention (vs. control) group, although the reduction in costs overall did not reach statistical significance (mean difference −£407.8 in intervention [vs. control] group, 95% CI −793.47 to +39.60).

The CEQ was completed by 357/403 (88.6%) participants, 177/201 (88.1%) in intervention and 180/202 (89.1%) in control groups (Figure S1). Responders were similar to the trial population overall (Table 1). On average, women were about 30 years of age, with ~20% from ethnic minority groups and over half with BMI ≥ 30 kg/m^2^ pre‐pregnancy or at antenatal care booking. Approximately half of women had chronic hypertension. Among the ~50% of women who were parous, ~15% had a prior Caesarean. The gestational age at randomisation was just over 37 weeks. Most women were on antihypertensive medication at enrolment—almost always one agent, which was usually labetalol. BP was controlled (< 140/90 mmHg) in ~75% of participants.

**TABLE 1:** Baseline characteristics of responders to Childbirth Experience Questionnaire, compared with the WILL trial population overall (*N*, % or mean [SD] unless otherwise stated).

	Responders (*N* = 357)	Trial population overall (*N* = 403)
Maternal age at randomisation (years)	31.9 (5.7)	31.7 (5.8)
Mother's self‐declared ethnicity		
White	279 (78.1%)	315 (78.2)
Black	26 (7.3%)	30 (7.4)
Arab	3 (0.8%)	3 (0.7)
South Asian	21 (5.9%)	25 (6.2)
Other/declined to give information	28 (7.8%)	30 (7.5)
Body mass index (kg/m^2^)		
< 18.5	0 (0)	0 (0)
18.5–24.9	61 (17.1%)	65 (16.1%)
25.0–29.9	89 (24.9%)	107 (26.6%)
≥ 30	126 (58.0%)	231 (57.3%)
Chronic hypertension^a^	173 (48.5)	198 (48.4%)
Gestational hypertension^a^	184 (51.5)	208 (51.6%)
Previous severe hypertension (sBP ≥ 160 mmHg or dBP ≥ 110 mmHg) during this pregnancy	40 (11.2)	42 (10.4%)
Pre‐gestational diabetes	4 (1.1%)	5 (1.3%)
Renal disease	9 (2.5%)	9 (2.2%)
Autoimmune disease (including APAS)	12 (3.4%)	14 (3.5%)
Nulliparous	172 (48.2%)	198 (48.1%)
In parous^b^ women	(*N* = 185)	(*N* = 209)
Prior Caesarean^a^	27/185 (14.6%)	31/209 (14.8%)
Prior gestational hypertension	105/185 (56.8%)	116/209 (55.8%)
Prior pre‐eclampsia^c^	47/185 (25.4%)	52/209 (25.0%)
Conceived by artificial reproductive technology^d^	14 (3.9%)	15 (3.7%)
Developed gestational diabetes in this pregnancy	33 (9.2%)	37 (9.2%)
Nicotine use after 20 weeks of current pregnancy	20 (5.6%)	23 (5.7%)
Taking low‐dose aspirin to prevent pre‐eclampsia	242 (67.8%)	268 (66.5%)
At trial enrolment		
GA at randomisation (weeks) (median [IQR])	37.1 [37.0, 37.4]	37.1 [37.0, 37.4]
BP and antihypertensive at enrolment		
Taking antihypertensive medication at consent	285 (79.8%)	321 (79.7%)
Taking one agent	264 (92.6%)	299 (93.2%)
Agents taken^e^	(*N* = 285)	(*N* = 321)
Labetalol	206/285 (72.3%)	234/321 (72.9%)
Nifedipine	68/285 (23.9%)	72/321 (22.4%)
Methyldopa	24/285 (8.4%)	28/321 (8.7%)
Other	9/285 (3.2%)	10/321 (3.1%)
Most recent sBP (mmHg) before consent	132.1 (10.5)	132.3 (10.6)
Most recent dBP (mmHg) before consent	83.0 (8.4)	83.3 (8.4)
Automated device used (any type)	254 (71.2%)	289 (71.7%)
Currently using home BP monitoring	204 (57.1%)	227 (56.3%)

Abbreviations: dBP, diastolic blood pressure; IQR, interquartile range; sBP, systolic blood pressure; SD, standard deviation; WILL, When to Induce Labour to Limit risk in pregnancy hypertension.

^a^ Minimisation variable, in addition to study site.

^b^ Number of previous deliveries of fetus at ≥ 22^+0^ weeks, ≥ 500 g birthweight, or a crown‐heel length ≥ 25 cm.

^c^ Pre‐eclampsia was defined as gestational hypertension with proteinuria or one or more relevant end‐organ complications (https://www.nice.org.uk/guidance/ng133).

^d^ Defined as in vitro fertilisation with or without intracytoplasmic sperm injection, donor egg, or donor sperm.

^e^ Responses are not mutually exclusive.

Responders gave birth in pre‐pandemic (67, 18.8%), pandemic with lockdowns (90, 25.2%) and pandemic without lockdown (200, 56.0%) periods, according to criteria by the United Kingdom government and The World Health Organization [15] (Figure S1); the distribution of births over these time periods was similar to all participants in WILL (i.e., 19.6%, 25.1%, 55.3%, respectively) (Figure S1).

### 3.1 Maternal Satisfaction With Care

In intervention (vs. control) groups, the median time to CEQ completion was 3 days earlier (Table 2). There was no evidence of a significant difference in CEQ scores overall, or by domain. Scores for ‘professional support’ were highest.

**TABLE 2:** Childbirth Experience Questionnaire findings for responders in the WILL trial (mean ± SD or *N*, % participants unless otherwise stated).

Outcomes	Planned early term birth at 38^+0–3^ weeks (*N* = 177)	Usual care at term (*N* = 180)	Mean difference^a^ (95% CI)
CEQ completion			
Days to completion (median [IQR])	2.0 [1.0, 13.0]	5.0 [1.0, 27.5]	NA
Completed before leaving hospital	98 (55.4%)	76 (42.2%)	NA
CEQ scores			
Total score	3.1 ± 0.4	3.1 ± 0.4	0.06 (−0.03 to 0.14)
Domains			
Own capacity	2.8 ± 0.5	2.7 ± 0.5	0.10 (−0.001 to 0.21)
Professional support^c^	3.7 ± 0.5	3.7 ± 0.6	0.04 (−0.08 to 0.16)^b^
Perceived safety	3.2 ± 0.6	3.1 ± 0.6	0.11 (−0.01 to 0.23)^b^
Participation^d^	(*N* = 161) 2.6 ± 0.7	(*N* = 161) 2.7 ± 0.6	−0.07 (−0.21 to 0.08)

Abbreviations: CEQ, Childbirth Experience Questionnaire; IQR, interquartile range; NA, not applicable.

^a^ Mean difference was calculated as intervention minus control, adjusted for minimisation variables, with centre as a random effect.

^b^ Centre was excluded as a covariate due to lack of convergence of the model.

^c^ For the question about whether the midwife devoted enough time to the woman's partner, 21 women, 5.9% (9 in intervention and 12 in the control group) responded that this question was ‘not applicable’.

^d^ The Participation subscale score was calculated only among women who did not have an elective Caesarean.

Table 3 presents CEQ responses overall and by domain, according to labour initiation and the main trial outcomes. Most women in each trial arm were induced, although the induction rate was higher in the intervention (vs. control) arm. Among those who were induced, and those with ‘poor maternal outcome’ (maternal co‐primary), neonatal care unit admission for ≥ 4 h (neonatal co‐primary), or Caesarean birth (key secondary outcome), CEQ scores were similar in intervention and control arms. Although pre‐eclampsia occurred significantly less often in the intervention (vs. control) group [9], CEQ scores were similar.

**TABLE 3:** Childbirth Experience Questionnaire response scores in the WILL trial, according to labour induction and outcomes (mean (SD) or *N*, % unless otherwise stated).

	Planned early term birth at 38^+0–3^ weeks (*N* = 177)		Usual care at term (*N* = 180)	
	Outcome	No outcome	Outcome	No outcome
Labour induction	(*N* = 153, 86.4%)	(*N* = 24, 13.6%)	(*N* = 123, 68.3%)	(*N* = 57, 31.7%)
Overall	3.1 (0.4)	3.4 (0.4)	3.1 (0.4)	2.9 (0.6)
Own capacity domain	2.7 (0.5)	3.1 (0.3)	2.7 (0.5)	2.6 (0.6)
Professional support domain	3.7 (0.5)	3.8 (0.5)	3.8 (0.4)	3.5 (0.8)
Perceived safety domain	3.2 (0.6)	3.4 (0.4)	3.2 (0.5)	3.0 (0.7)
Participation domain^a^	(*N* = 153) 2.6 (0.7)	(*N* = 8) 2.7 (0.6)	(*N* = 122) 2.7 (0.6)	(*N* = 39) 2.5 (0.7)
Spontaneous onset of labour	(*N* = 8, 4.5%)	(*N* = 169, 95.5%)	(*N* = 40, 22.2%)	(*N* = 140, 77.8%)
Overall	3.1 (0.3)	3.1 (0.4)	2.8 (0.5)	3.1 (0.4)
Own capacity domain	2.9 (0.3)	2.7 (0.5)	2.4 (0.5)	2.7 (0.5)
Professional support domain	3.7 (0.6)	3.7 (0.5)	3.5 (0.8)	3.8 (0.5)
Perceived safety domain	3.2 (0.3)	3.2 (0.6)	2.9 (0.7)	3.2 (0.5)
Participation domain^a^	(*N* = 8) 2.7 (0.6)	(*N* = 153) 2.6 (0.7)	(*N* = 39) 2.5 (0.7)	(*N* = 122) 2.7 (0.6)
‘Poor maternal outcome’	(*N* = 26, 14.7%)	(*N* = 151, 85.3%)	(*N* = 22, 12.2%)	(*N* = 158, 87.8%)
Overall	3.0 (0.4)	3.1 (0.4)	3.0 (0.4)	3.1 (0.4)
Own capacity domain	2.7 (0.5)	2.8 (0.5)	2.7 (0.6)	2.6 (0.5)
Professional support domain	3.7 (0.5)	3.8 (0.5)	3.6 (0.8)	3.7 (0.6)
Perceived safety domain	3.2 (0.6)	3.3 (0.6)	3.1 (0.5)	3.1 (0.6)
Participation domain^a^	2.4 (25, 0.7)	2.6 (136, 0.7)	2.4 (19, 0.7)	2.7 (142, 0.6)
Neonatal care unit adm ≥ 4 h	(*N* = 14, 7.9%)	(*N* = 163, 92.1%)	(*N* = 12, 6.7%)	(*N* = 168, 93.3%)
Overall	3.1 (0.4)	3.1 (0.4)	2.9 (0.5)	3.1 (0.4)
Own capacity domain	2.7 (0.5)	2.8 (0.5)	2.4 (0.6)	2.7 (0.5)
Professional support domain	3.8 (0.4)	3.7 (0.5)	3.8 (0.4)	3.7 (0.6)
Perceived safety domain	3.2 (0.8)	3.2 (0.5)	2.8 (0.5)	3.2 (0.6)
Participation domain^a^	(*N* = 13) 2.5 (0.7)	(*N* = 148) 2.6 (0.7)	(*N* = 11) 2.5 (0.7)	(*N* = 150) 2.7 (0.6)
Caesarean birth	(*N* = 50. 28.2%)	(*N* = 127, 71.8%)	(*N* = 65, 36.1%)	(*N* = 115, 63.9%)
Overall	3.1 (0.5)	3.1 (0.4)	3.0 (0.4)	3.1 (0.4)
Own capacity domain	2.7 (0.6)	2.8 (0.5)	2.6 (0.6)	2.7 (0.5)
Professional support domain	3.7 (0.5)	3.8 (0.5)	3.7 (0.5)	3.7 (0.6)
Perceived safety domain	3.2 (0.5)	3.3 (0.6)	3.0 (0.6)	3.2 (0.6)
Participation domain^a^	(*N* = 34) 2.3 (0.6)	(*N* = 127) 2.7 (0.7)	(*N* = 47) 2.6 (0.6)	(*N* = 114) 2.7 (0.7)
Pre‐eclampsia	(*N* = 49, 27.7%)	(*N* = 128, 72.3%)	(*N* = 67, 37.2%)	(*N* = 113, 62.8%)
Overall	3.0 (0.4)	3.1 (0.4)	3.0 (0.4)	3.1 (0.4)
Own capacity domain	2.7 (0.5)	2.8 (0.5)	2.6 (0.5)	2.7 (0.5)
Professional support domain	3.7 (0.6)	3.8 (0.5)	3.6 (0.7)	3.8 (0.5)
Perceived safety domain	3.2 (0.5)	3.3 (0.6)	3.1 (0.6)	3.2 (0.6)
Participation domain^a^	(*N* = 46) 2.6 (0.7)	(*N* = 115) 2.6 (0.7)	(*N* = 63) 2.6 (0.7)	(*N* = 98) 2.7 (0.6)

Abbreviation: adm, admission.

^a^ The Participation subscale score was only calculated among women who did not have an elective Caesarean birth.

Table 4 presents the summary of directed content analysis of 378 free‐text comments made by 93/177 (52.5%) participants in the intervention group and 98/180 (54.4%) in the control group. Thirteen other comments were uninformative and excluded (seven in intervention and six in control). Comments were sorted into the four CEQ themes (i) ‘Capacity for autonomy over care’ (corresponding to CEQ domain: ‘Own Capacity’); (ii) ‘Relational care and care interactions’ (CEQ domain: ‘Professional support’); (iii) ‘Conceptualising safety’ (CEQ domain ‘Perceived safety’); (iv) ‘Lack of shared decision‐making’ (CEQ domain: ‘Participation’), and two additional themes, (v) ‘Other experiences of labour and birth’; and (vi) ‘Experience of participating in research’. Table S3 shows examples of free text comments for each of the themes.

**TABLE 4:** Directed content analysis of free‐text comments by Childbirth Experience Questionnaire responders^a^ (*N*, % responders).

Themes	Planned early term birth at 38^+0–3^ weeks (*N* = 93)^b^		Usual care at term (*N* = 98)^b^	
	TOTAL positive (*N* = 111)	TOTAL negative (*N* = 75)	TOTAL positive (*N* = 111)	TOTAL negative (*N* = 81)
Relational care and care interactions (CEQ ‘Professional support’ domain) *N* = 160 responders	Positive (*N* = 64)	Negative (*N* = 17)	Positive (*N* = 62)	Negative (*N* = 17)
	My midwife was amazing, she made me and my partner feel so safe and happy. My care couldn't have been better Very happy with care received. The whole team was fantastic, everyone was positive	I felt due to the inducing process not working as planned: as afetr [after] the first gel insertion, the baby went bradycardic for over 5 min this then put me off having the next 2 gels as planned. I felt 1 particular senior midwife felt that I was been difficult not wanting the gels and treat me inappropr [inappropriately] Midwives repeatedly telling me to wait for pain relief and wait even further for an examination due to being busy on rounds	I received amazing care, I felt my dignity was guarded, and felt in safe hands at all times The team were brilliant and were so reassuring I felt we were both in very capable and professional hands. All in all we had a wonderful delivery—Thank you!	I was left to labour in the assessment room on my own with no midwife. The baby's head came out before anyone came to me. I had no midwifery care in labour. Precipitate labour (45 min). Husband also felt traumatised by this experience Midwife should listen to patients more
Capacity for autonomy over care experiences (CEQ ‘Own capacity’ domain) *N* = 55 responders	Positive (*N* = 2)	Negative (*N* = 24)	Positive (*N* = 5)	Negative (*N* = 24)
	I had an epidural and required on [an] episiotomy, both were discussed with me prior to these, and risks and benefits explained overall a very positive experience	Induction took a lot longer than I expected given it was my second child. This made me lose confidence at times though the midwives helped keep me going I felt out of control due to the additional pain and how things needed to escalate to the C‐section which was required	I felt completely in control until circumstances in labour meant an emergency c‐section was indicated. All the staff respected my wishes and accommodated them wherever possible	Unhappy with care in labour. Midwives kept sending me home telling me I was not in labour. Would not give me any pain relief until I was 4 cm. I was in too much pain in my back I feel I could have received an epidural from the moment I asked for one—I had to wait hours to get one. My experience in X ward was horrible but once I got into the labour suite and got the epidural I had excellent service from the midwives and they treated me so well
Conceptualising safety (CEQ ‘Perceived safety’ domain) *N* = 68 responders	Positive (*N* = 25)	Negative (*N* = 10)	Positive (*N* = 23)	Negative (*N* = 10)
	Our birth experience changed very quickly but once we were heading to theatre, despite the frantic nature, I felt safe and secure. Initially on the IOL ward the midwife seemed quite stretched and was very helpful when she was with you, but some hard to get hold of I cannot thank the midwives enough for helping me through labour and making sure I was ok throughout it. My blood pressure was well kept controlled thanks to the midwives	I would have liked to be examined how dilated I was when my contractions got closer. As someone who had a 3A (third degree) tear I felt I was being told by the midwives to push a bit too soon and I told them no I am not pushing, I actually held back pushing as I was so scared to tear I felt frightened and thought something was wrong even though I was told it was okay. I felt neglected as I was not given Gas & Air when I asked for it. Induction was not as I imagined. I was terrified during the induction	It was quite a traumatic experience for me, but I felt well informed and felt in very safe hands all the way through I can't thank my midwife and the whole team at the X for the safe delivery of my baby boy. X my midwife was incredible and i felt so safe with her during such a scary time Although ended up not as expected all care given was very supportive and I felt everyone was in control and made everything feel calm	Conflicting information about dilatation in labour made for a more traumatic birth experience It seemed that the diagnosis of pre‐eclampsia was only made at the point when they did the caesarean [Caesarean]. My organs were swollen and the doctor said she wouldn't have delivered normally
Lack of shared decision‐making (CEQ ‘Participation’ domain) *N* = 12 responders	Positive (*N* = 0)	Negative (*N* = 5)	Positive (*N* = 0)	Negative (*N* = 7)
	—	This was a different labour for me: the drip & monitor made it very uncomfortable and I would have liked to be able to move around	—	Found it very hard to answer some questions due to labour happening very quickly, overall found it quite intense. Was all quite a lot to take in after being told it was potentially false labour as this was my first child. Due to guidance as well was no time to discuss/change position or have any pain relief
Other experiences of labour and birth *N* = 64 responders	Positive (*N* = 12)	Negative (*N* = 16)	Positive (*N* = 16)	Negative (*N* = 20)
	Delighted in every way with the way things happened Although my birth didn't go to plan (ended with EMCS) I was happy with the care I received and could not be happier with the experience	Felt that experience with delivering the placenta negatively impacted birth experience I was not induced in the end. After sweep on Friday X I was 2 cm dilated. The ANW did not want to use other methods to speed up labour. Labour ward was extremely full. 2 days later my waters broke and I went to full contractions every minute. There was no time for pain relief which made the … experience very challenging, scary and negative. No time for options	Overall a great experience All in all we had a wonderful delivery—Thank you!	It wasn't a good experience, the whole pregnancy wasn't a good experience Had hormone drip used this was brutal and if given the option of it I wouldn't like to have it again. Because of it I felt in too much pain all of a sudden instead of steadily
Experience of participating in research *N* = 19 responders	Positive (*N* = 8)	Negative (*N* = 3)	Positive (*N* = 5)	Negative (*N* = 3)
	Glad I was induced when I was, happy to get earlier induction on the WILL trial Happy that she participated because when she came in to deliver on the induction date, the team found CTG concerns if it was not for the study she may not have had a safe delivery of her baby	I feel the whole process was a shambles I came in on Thursday X, did not have my baby until X. Please do not induce women at 38 weeks with hypertension. There is nothing to gain, I will never have another child at the X due to this experience. All I have taken away as a participant is a heap load of physical mental and unnecessary stress. Please do not induce babies that aren't ready Induction date was Wednesday X. They couldn't get me a bed on the labour ward until Monday X. I was made to stay in the hospital for 5 days waiting for a bed. Really bad experience. Would not recommend, as induction date is not guaranteed	Happy to have participated in research study This woman had a very positive child birth experience compared to her last birth. Was happy participating in the WILL study	I really felt my induction should have been much earlier due to my Baby being predicted as big. But even at a scheduled 39 weeks induction I did not have my baby until 39 + 6 because of how busy the hospital was I strongly believe a way should be made possible for women who agree to take part in this trial so they do not wait as long as I did. I felt frustrated and anxious at some point because of the many hours of waiting for a bed to break my waters to start my induction

Abbreviation: CEQ, Childbirth Experience Questionnaire.

^a^ Categories are not mutually exclusive.

^b^ *N* = 13 comments were not included in the analysis, as they were uninformative: ‘Everything was Fine’; ‘Some questions difficult to answer in view of elective LSCS (“I felt I could have a say whether I could get up and about or lie down,” “I felt I could have a say in deciding my birth position”)’; ‘Thank you’; ‘No comment’; ‘Nothing else to share’; ‘No’; ‘No further comments to add’; ‘N/A’; ‘I had an elective section so some of these questions are not applicable’; ‘no additional comments’; ‘scores are based on caesarean section delivery’; ‘nothing to add’; ‘This lady had elective CS and did not feel able to answer the questions with missing answers’.

The majority of comments were positive (222/378, 58.7%), and neither their number nor nature (positive or negative) appeared to differ between intervention and control groups. Representative quotations are presented in Table 4.

The majority of comments were attributed to the theme of ‘Relational care and care interactions’ (160/378, 42.3% of comments), and were positive (126/160, 78.8%) (Table 4). In particular, women thanked staff (especially midwives) for the care received. Nevertheless, some described negative experiences, particularly not feeling listened to regarding pain relief, or having their decisions respected.

Three themes were supported by a similar number of comments: ‘Capacity for autonomy over care experiences’ (*N* = 55), ‘Conceptualising safety’ (*N* = 68) and ‘Experiences of labour and birth’ (*N* = 64).

Most comments related to ‘Capacity for autonomy over care experiences’ were negative (*N* = 48/55, 87.3%), reflecting an induction process which: took longer than anticipated, felt ‘out of control’, or was more painful, with that pain not addressed adequately by staff (Table S3).

Most comments about ‘Conceptualising safety’ were positive (48/68, 70.6%) (Table 4), even when plans took an unexpected turn (e.g., emergency Caesarean), and were often related to perceived good communication. Nevertheless, there were a substantial number of negative comments, related to: fear that induction was not going as they had anticipated, not being fully reassured, receiving conflicting information, not being listened to or supported, being asked to do something (e.g., push) with which they were uncomfortable, or being sent home and told that they were not in labour.

Comments related to the ‘Experiences of labour and birth’ were a fairly even mix of positive and negative (Table 4). Positive comments were fairly general. Negative comments reflected on overall pregnancy experience or aspects of general management of labour, such as pain related to rapid onset of contractions or labour augmentation using oxytocin (‘*hormone drip*’).

Few women made comments related to ‘Lack of shared decision‐making’ (*N* = 12), but when they did, all were negative, related to medicalisation of birth initiation, and inadequate time available to discuss options with rapid onset of labour (Table 4).

Few women made comments about their ‘Experience of participating in research’ (*N* = 19). Most comments were positive, and included acknowledgement by women that they appreciated the opportunity to be induced earlier which resulted in problems being detected that they felt might not have otherwise been, or were related to a positive experience overall. Negative comments noted problems with initiating the intervention (of early term birth by labour induction), and questions about the advisability of expectant care.

## 4 Discussion

### 4.1 Main Findings

In this trial of 403 high‐risk women with chronic or gestational hypertension, randomisation to planned early term birth at 38^+0–3^ weeks (vs. usual care at term) resulted in a similar childbirth experience overall, by CEQ domain, and whether labour induction or adverse outcomes had occurred.

Just over half of women who responded to the CEQ provided free‐text comments, and most were positive, particularly regarding ‘Relational care and care interactions’ (CEQ ‘Professional support’ domain). Also, most comments endorsed the ‘Conceptualising safety’ theme, even when labour induction did not go as planned. However, it was clear that labour induction was not always viewed positively, in both arms and across themes of ‘Capacity for autonomy over care’, ‘Experiences of labour and birth’, and the less frequently‐endorsed themes of ‘Lack of shared decision‐making’ and ‘Experience of participating in research’. Repeatedly, women described logistical issues related to initiation of labour induction, lack of information, the process not going as they had expected, uncontrolled pain, and not always being listened to.

### 4.2 Interpretation

The CEQ is particularly important in trials of timed delivery, because it assesses the maternal perspective on the birthing experience, including satisfaction and psychological impact, beyond clinical outcomes, like mode of birth or neonatal health. The UK National Institute for Health and Care Excellence (NIHR) mandates that women's experiences be measured in maternity intervention studies that they support, and the CEQ does so in a standardised and reproducible way. Feedback was received from the vast majority of WILL participants, at a response rate higher than seen in other studies using the CEQ (59%–78%) [12, 13, 16, 17, 18]. Although in an unblinded trial, the intervention is anticipated to improve safety, and women in the control group may experience heightened anxiety, there was no difference in satisfaction between groups.

It is likely that professional support consistently scored highest given that respondents were all participants in a randomised trial, and thus, had more discussion about their options and in this trial, more support during their care near and at term gestational age. This serves to emphasise the positive experiences of those who participate in research.

In each trial arm, most women underwent labour induction, but despite more doing so in the intervention (vs. control) arm, CEQ scores were similar. While labour induction has been associated with a more negative childbirth experience [19, 20, 21], many such studies compared labour induction with spontaneous labour onset; however, women cannot choose the latter, but can only choose to wait for it, in the hope that an indication for timed birth (by labour induction, or elective Caesarean) will not arise first. It was the direct comparison of labour induction with spontaneous onset of labour that led to erroneous conclusions that labour induction increases Caesarean, when in fact, induction appears to *reduce* it compared with expectant care [22]. While it was clear that labour induction was not viewed positively in the WILL trial, as has been published by others [23], the free‐text comments made by women can be used to improve induction experiences, such as: good information‐sharing and preparation which facilitate a sense of ownership and control of labour [24], and which we reported earlier could be improved within NHS labour induction patient information materials [25].

Women who participated in WILL were high‐risk, but they did not have more negative childbirth experiences [26, 27, 28]. In intervention studies of planned birth (vs. ongoing expectant care), CEQ scores were similar, for nulliparous women with advanced maternal age [17] and those with post‐term pregnancy [29]. The exception was more favourable participation scores in the intervention group in the post‐term trial [29]. As in other intervention trials [19, 29], we observed the highest domain score for ‘Professional support’ (range 3.5–3.7), and the lowest for ‘Own capacity’ (range 2.5–3.0) and ‘Participation’ (range 2.6–2.9). ‘Professional support’ was the theme commented on most frequently, and those comments were overwhelmingly positive, a point worth emphasising given low morale in United Kingdom maternity services [30].

The CEQ used was the version available when the WILL trial began [12, 13]. CEQ2 has now been developed [31, 32]. ‘Participation’ was reworked with new items related to information‐sharing and decision‐making, mirroring the free‐text comments made by WILL participants, which led us to rename the ‘Participation’ theme to ‘Lack of shared decision‐making’. All comments were negative, supporting revision of the CEQ, from one focussed on labour position, mobility and pain relief method, to information exchange and shared responsibility for management decisions.

### 4.3 Strengths, Limitations

Strengths include the high‐quality conduct of the WILL trial and recruitment from 42 United Kingdom hospitals serving populations with broad demographics. Participants gave birth over pre‐pandemic to post‐pandemic time periods, so our results are generalisable. We used the CEQ, one of the most commonly‐used measures of the multidimensional experience of labour and birth, and one validated for use in the United Kingdom [33]; our response rate was high for a postnatal questionnaire. An evaluation of free‐text comments was included to capture dimensions of childbirth experience not well‐explored by multiple choice questions.

Limitations include that women in the intervention (vs. control) group completed the CEQ a few days earlier, but the original CEQ was validated for use within one month following birth [12, 13], by which time the vast majority of women in both trial arms had completed it. Second, we did not measure the method of CEQ completion, and it is likely that more women in the control (vs. intervention) arm completed the questionnaire after discharge, either self‐administered and posted, or over the phone with study teams. This may have been due to the higher likelihood of planned, controlled birth in the intervention group, compared to the unplanned nature that occurred more often in the control group. Women may have been concerned about research staff reading negative comments, even though they did not provide clinical care. Nevertheless, there were no between‐group differences in CEQ answers or free‐text comments. Third, the CEQ was designed to examine experiences after the first childbirth; while approximately half of women in WILL were parous, the CEQ is considered acceptable for use in these women. Fourth, the experiences of women who participated in the trial may be different from those who chose not to. There are aspects of the CEQ that are not applicable to all women; examples include the ‘Participation’ domain for women who chose an elective Caesarean birth; those without a partner; those who received care from multiple members of staff with whom they had different experiences; or those whose mobility changed during labour. Also, it is possible that staff treated women in the trial differently, knowing that their childbirth experience would be measured. Finally, the CEQ was provided only in English, although the ethnic mix of women was similar in intervention and control groups.

## 5 Conclusions

For women with chronic or gestational hypertension who remain well at term gestational age, we found no difference in childbirth experience between women randomised to planned early term birth at 38^+0–3^ weeks' gestation, compared with usual care at term. This was true regardless of initiation of birth, mode of birth, or pregnancy outcome, and in directed content analysis of free‐text comments. Based on these findings, shared decisions about the timing of birth may be more influenced by differences in clinical outcomes and costs. Additionally, labour induction experiences may be improved with good information‐sharing and preparation, to facilitate a sense of ownership and control of labour.

## Author Contributions

The trial was conceived by L.A.M., J.A.H., J.S., and P.vD. All authors contributed to the trial's design and delivery. C.A.M. and E.G. assume responsibility for the accuracy and completeness of data analysis and reporting. L.A.M., K.K., and S.T. vouch for the analysis's fidelity to the protocol. S.T., K.K., and L.A.M. drafted the manuscript. All authors revised and approved it.

## Ethics Statement

The trial was approved (10/Jan/2019) by the NHS Health Research Authority London Fulham Research Ethics Committee (reference 18/LO/2033).

## Conflicts of Interest

The authors declare no conflicts of interest.

## Supporting information


**Figure S1.** Timing of birth for all women randomised in WILL, according to pre‐pandemic and pandemic epochs.
**Table S1.** The WILL Trial Study Group.
**Table S2.** Childbirth Experience Questionnaire 1.
**Table S3.** Directed content analysis of free‐text comments by Childbirth Experience Questionnaire responders* (*N*, % responders).

## Data Availability

Requests for data should be directed to (bctudatashare@contacts.bham.ac.uk). Requests will be assessed for scientific rigour before being granted. Data will be anonymised and securely transferred. A data‐sharing agreement might be required.
