# Supplementary material for: Women's Childbirth Experiences in the WILL Randomised Trial (When to Induce Labour to Limit Risk in Pregnancy Hypertension): A Mixed Methods Analysis
Source: BJOG. 2025 Jun 24;132(10):1426–37. doi: 10.1111/1471-0528.18257 (PMC12315064; doi:10.1111/1471-0528.18257)

**SUPPLEMENTARY APPENDIX**

| **Document** | **Title** | **Page number** |
| --- | --- | --- |
| **Tables** |  |  |
| Table S1 | WILL Trial Study Group | 2 |
| Table S2 | Childbirth Experience Questionnaire 1 | 5 |
| Table S3 | Directed content analysis of free-text comments by Childbirth Experience Questionnaire responders | 7 |
| **Figures** |  |  |
| Figure S1 | Timing of birth relative to pre-pandemic and pandemic epochs, for all women randomised in WILL | 12 |

**Table S1**: The WILL Trial Study Group

| **From Clinical Investigator, Co-investigator and Trial Management Groups** |
| --- |
| Laura A. Magee (Chief Investigator), Peter Brocklehurst, Lucy Chappell, Sean Cole, Jon Dorling, Ruth Evans, Max Feltham, Eleni Gkini, Marcus Green (PPIE), Pollyanna Hardy, Jennifer Hutcheon, Katie Kirkham, Lisa Leighton, Catherine Moakes, Ben Mol, Katie Morris, Mary Nulty, Paul Riley, Jesse Kigozi, Janet Scott (PPIE), Joel Singer, Clive Stubbs, Kiran Sunner, Jim Thornton, Sue Tohill, Peter von Dadelszen, Julie Wade |
| **Trial Management Group** |
| Laura A. Magee (Chief Investigator), Peter von Dadelszen, Eleni Gkini, Katie Kirkham, Catherine Moakes, Paul Riley, Clive Stubbs, Jim Thornton, Sue Tohill |
| **Clinical Investigator Group** |
| Laura A. Magee (Chief Investigator), Peter Brocklehurst, Lucy Chappell, Peter von Dadelszen, Jon Dorling, Pollyanna Hardy, Jennifer Hutcheon, Ben W. Mol, Tracy Roberts, Janet Scott, Joel Singer, Jim Thornton |
| **From WILL trial sites** |
| **Airedale General Hospital:** Sumita Bhuiya, Soumendra Nallapeta, Emma Dooks, Sophie Packham, Chantal McParland |
| **Birmingham Women's Hospital**, Birmingham: Diane Whitehouse, Chloe O'Hara, Connie Weston, Diane Mellers, Lesley Brittain, Phern Adams, Katie Morris, Rebecca Shakespeare, Emily Pain, Natalie Bayne, Sethenia Beckford, Sunena Verma |
| **Bradford Teaching Hospitals NHS Foundation Trust**, Bradford: Sudeepthi Kakara, Janet Wright, Amal Mighell, Jennifer Syson, Kari Swettenham, Shaila Seraj, Georgina Goodaire, Jenny Butler, Kate Pittendreigh, Liz Ingram, Hannah Brooks |
| **Cardiff and Vale University Health Board:** Maryanne Bray, Claire Bertorelli, Hannah Ritter, Emma Pugh |
| **Chesterfield Royal Hospital:** Janet Cresswell, Mary Kelly-Baxter, Li-Shan Yeoh, Shailly Sahu Bhansali, Vandana More, Fiona Warburton, Lauren Bishop, Nikit Kadam, Shefali Rathee, Deepika Goyal |
| **Croydon University Hospital**, Croydon: Bini Ajay, Geraldine Upson, Danielle Hake, Diana Opoku, Emma Wayman, Natalia Cwiek, Stacy Tregellas, Nikki Lee |
| **Cwm Taf Morgannwg UHB:** Lavinia Margarit, Joelle Pike, Kate Jones, Sophie-Mae Wheeler-Davies, Meena Ali, Vikki Drake, Deborah Jones |
| **East Surrey Hospital:** Indhuja Rajkumar, Ruth Habibi, Sarah Davies, Emma Crawley, Katie Prickett, Kopal Agarwal, Rebecca Low, Sumit Kar, Kate Stringer |
| **Glangwili Hospital:** Harinakshi Salian, Trudy Smith, Anangsha Kumar |
| **The James Cook University Hospital**, Middlesbrough: Deepika Meneni, Hazel Alexander, Helen Harwood, Kerry Hebbron, Lynn Whitecross, Mary Hodgers, Shilpa Mahadasu, Rebecca Fletcher |
| **King's College Hospital:** Nick Kametas, Yasmin Sana, Hayley Martin, Rebecca Jarman, Sophie Webster, Gillian Godwin, Leia Parry, Naomi Grimes |
| **King’s Mill Hospital:** Jyothi Rajeswary, Mandy Gill, Emily Omuvwie, Rachel Johnson, Susan Smith |
| **Kingston Hospital:** Gabrielle Bambridge, Danielle Hake, Isabel Bradley, Kristina Sexton, Lola Oshodi, Kathryn Sollesta |
| **University Hospitals of Leicester NHS Trust**, Leicester: Cornelia Wiesender, Claire Dodd, Rupa Modi, Beverley Cowlishaw, Gina Mulheron, Magdalena Kierzenkowska, Molly Patterson, Patricia Amos, Sharon Marie Bates, Sharon Raper, Anna Holt, Sarah Evans |
| **Liverpool Women’s Hospital**, **Liverpool:** Umber Agarwal, Ruth Cockerill, Amy Mahdi, Caroline Cunningham, Michelle Dower, Sian Rogers, Siobhan Holt, Carly Williams, Zora Castling, Linda Watkins, Rachel McFarland |
| **New Cross Hospital, Wolverhampton:** David Churchill, Ellmina McKenzie, Julie Icke, Laura Devison, Lindsey Jarosz, Philippa Rafferty |
| **North West Anglia NHS Foundation Trust, Peterborough City Hospital and Hinchingbrooke Hospital (closed):** Sreejith Kodakkattil, Charleen Lia, Coralie Huson, Jodi Carpenter, Kimberley Morris |
| **Northumbria Specialist Emergency Care Hospital:** Vinita Raheja, Angela Ayuk, Jessica Reynolds, Julie Wyton, Stacey Duffy |
| **Northwick Park Hospital:** Sahana Gupta, Anam Fayadh, Sean Connarty |
| **Nottingham City Hospital**, Nottingham**:** Kate Walker, Jim Thornton, Jane Cantliffe, Catriona Hussain, Carys Smith, Harriet Anderson, Lesley Hodgen, Megan Betteridge, Nahid Kazmi,Yvette Davis |
| **Princess Anne Hospital,** Southampton: Karen Brackley, Nicki Martin Fiona Walbridge, Rhea Hampton, Alexandra Kermack, Kate Raney |
| **Queen Elizabeth Hospital King’s Lynn:** Salman Kidwai, Aricsa Joshy, Hollie Curgunven, Esther Dorken, Uma Stephen Paul |
| **Queen's Medical Centre**, Nottingham: Kate Walker, Jim Thornton, Catriona Hussain, Jane Cantliffe Carys Smith, Lesley Hodgen, Nia Jones, Julie Shaw, Megan Betteridge, Nahid Kazmi, Yvette Davis |
| **Raigmore Hospital (Highland):** Charlotte Barr, Deborah McDonald, Donna Patience, Jandy Fernandes, Sadia Akhtar, Shona Macleod |
| **Royal Berkshire Hospital:** Patrick Bose, Catherine Young, Fidelma Lee, Anna Campbell, Sharon Westcar |
| **Royal Bolton Hospital (closed):** Rebecca Peart, Emma Tanton, Kat Rhead |
| **Royal Cornwall Hospital:** Kristin Fiedler, Ruth Bowen, Richard Keedwell |
| **Royal Oldham Hospital:** Stephy Mathen, Zainab Sarwar, Chloe Rishton, Chloe Scott, Marcus Cabrera-Dandy, Grainne O’Connor |
| **Royal United Hospital:** Bath (closed): Jane Farey, Nisha Verasingam, Mel Rich, Annette Moreton, Catherine Bressington, Jennifer Pullen, Sara Burnard, Wendy Duberry, Jessica Sellick |
| **Singleton Hospital**, Swansea: Madhuchanda Dey, Sharon Jones, Pauline Bird, Joelle Morgan, Charu Gupta |
| **South Tyneside General Hospital & Sunderland Royal Hospital**, South Tyneside & Sunderland NHS Foundation Trust, Sunderland: Aarti Ullal, Eileen Walton, Ashleigh Price, Janet Scollen, Judith Ormonde, Kirsten Herdman, Lesley Hewitt, Lucy Rowland |
| **Southend University Hospital:** Mandeep Singh, Sundararajah Raajkumar, Beena Saji |
| **St George's University Hospital:** Asma Khalil, Alice Perry, Emily Marler, Ijeoma Imuzeze, Sophie Robinson, Lolade Oshodi, Hafiza Koroma |
| **St James’s University Hospital,** The Leeds Teaching Hospitals NHS Trust, Leeds: Jonathan Nelson, Kathryn McNamara, Carina Craig, Del Endersby, Jayne Wagstaff, Kate Robinson, Hannah Barnes, Jane Gavin, Emily Scriven, Peter Owegie, Linden Stocker |
| **St Mary’s Hospital, Manchester**: Jenny Myers, Kate Stanbury, Christine Hughes, Sarah Lee, Kate Duhig, Flurina Michelotti, Olivia Moran |
| **St Mary’s Maternity Unit, Poole Hospital,** University Hospitals Dorset NHS Foundation Trust: Latha Vinayakarao, Louise Melson, Stephanie Grigsby, Susara Blunden, Annemie Legg, Julie Beeson |
| **St Michael’s Hospital, Bristol:** Melanie Griffin, Sarah Newell, Katharine Jane Thompson, Brittany Smart, Elizabeth Payne, Marie Pitchford, Jing Lin |
| **St Richard’s Hospital & Worthing Hospital:** Rahila Khan, Sophia Stone, Ahmed Elgarhy, Emma Meadows, Marian Flynn-Batham, Nikky Passmore, Vivienne Cannons |
| **St Thomas’ Hospital (paused)**, Guy’s and St Thomas’ NHS Foundation Trust, London: Declan Symington, Alice Lewin, Hayley Tarft, Jessamine Hunt, Julie Wade, Sue Tohill, Zoe Vowles, Maria Slaney, Rachel Woodcock, Hilary Thompson, Lucy Chappell |
| **The Great Western Hospital, Swindon:** Alex Van der Meer, Tracey Benn, Ru Davies |
| **The Royal Victoria Infirmary, Newcastle:** Gareth Waring, Jill Riches, Andrea Fenn, Aly Kimber, Susan Harrop, Falak Diab, Angela Phillipson |
| **University College London Hospital:** Daniel Stott, Amos Tetteh, Davide Casagrandi, Miriam Bourke, Eirini Vaikousi, Rita Sarquis, Morenike Folorunsho, Olivia Newth, Sarah Weist, Yaa Acheampong, Vidhya Ravikumar, Adele Powell, Amos Tatteh, Anna David, Jennifer Tshibamba, Lauren Laville, Sarah Ekladios |
| **University Hospital North Tees:** Stephen Wild, Emily Slane, Julie Woollaston, Kirsty Farrington, Sharon Gowans, Vicky Collins |
| **University Hospital of North Durham:** Jemma Yorke, Vicki Atkinson, Shelly Wood |
| **University Hospital Wishaw (Lanarkshire):** Eleanor Jarvie, Carol Hollas, Denise Vigni |
| **Walsall Manor Hospital:** Tigist Mengistu, Robert Chadwick, Helen Haden, Lisa Richardson, Iwona Wojtuszko, Susan Musa |
| **West Middlesex University Hospital (closed)**: Middlesex: Joanna Girling, Amy Barker, Elaine Palmer, Louise Page, Grace Ryan, Lauren Trepte, Samantha Steele |
| **York Hospital (closed)**, York: Jacqueline Tang, Harriet Pearson, Jo Ingham, Nicola Spark, Samantha Roche, David Thompson |

**Table S2**: Childbirth Experience Questionnaire 1


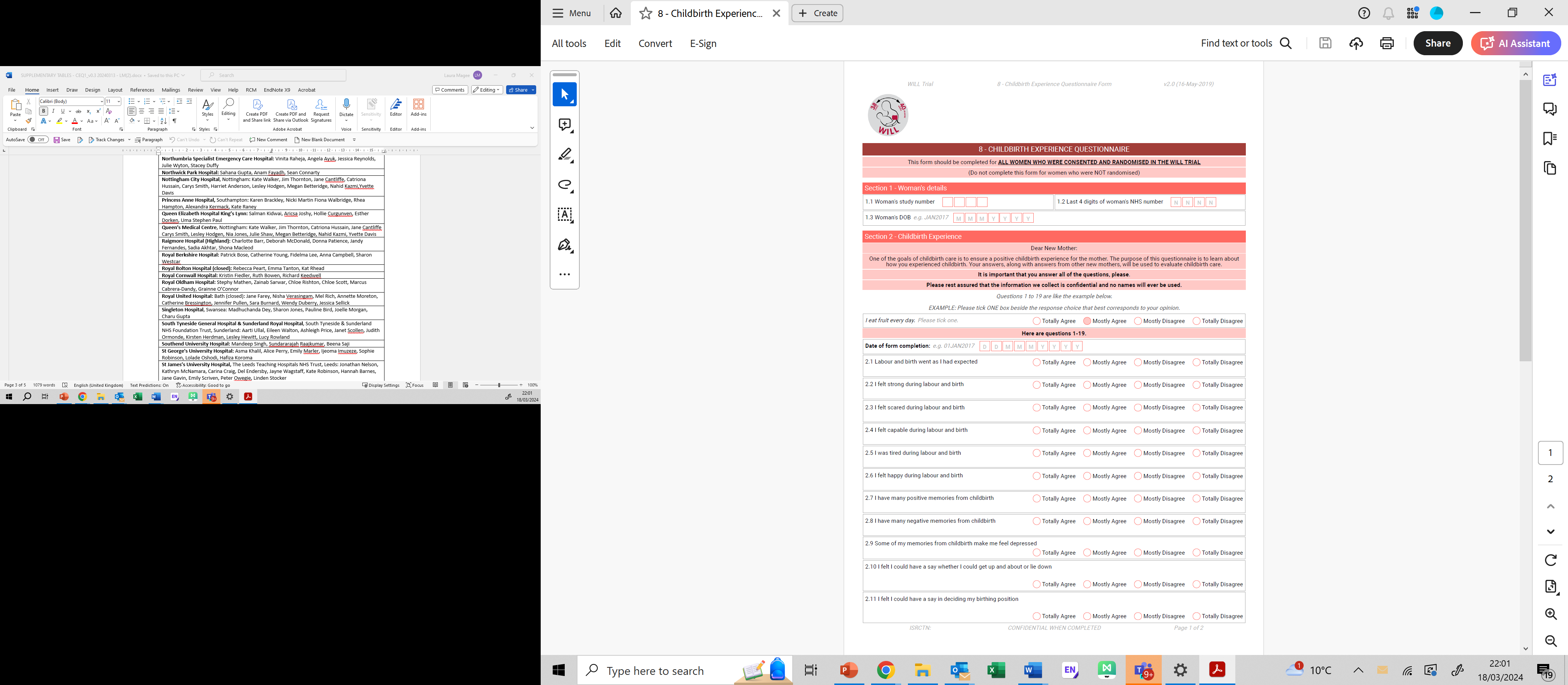


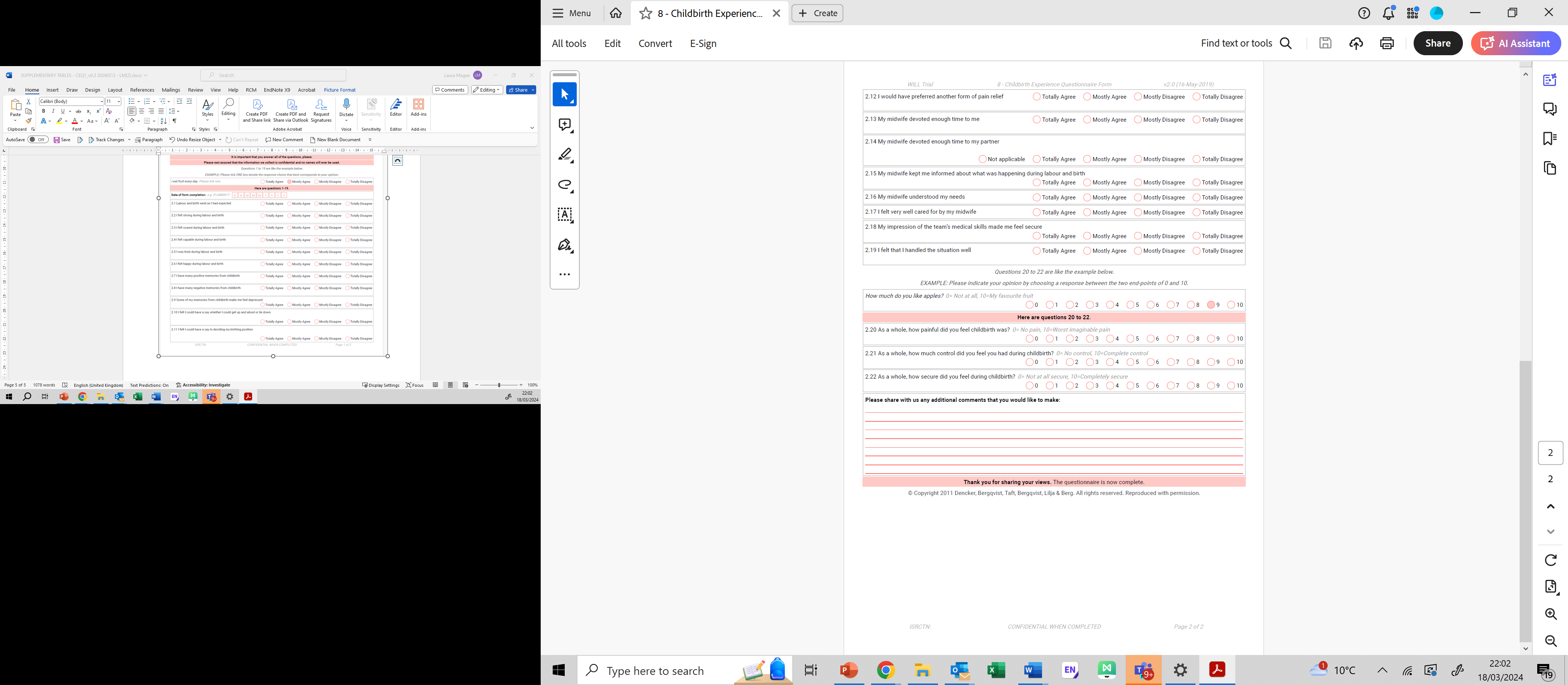


**Table S3**: Directed content analysis of free-text comments by Childbirth Experience Questionnaire responders* (N, % responders)

| **Themes** | **Planned early term birth at 38^+0-3^ wks** (N=93)† | | **Usual care at term** (N=98)† | |
| --- | --- | --- | --- | --- |
|  | TOTAL Positive (N=111) | TOTAL Negative (N=75) | TOTAL Positive (N=111) | TOTAL Negative (N=81) |
| **Relational Care and Care Interactions**  (CEQ ‘Professional support’ domain)  N=160 responders | Positive (N=64) | Negative (N=17) | Positive (N=62) | Negative (N=17) |
|  | My midwife was amazing, she made me and my partner feel so safe and happy. My care couldn't have been better.  Very happy with care received. the whole team was fantastic, everyone was positive. | I felt due to the inducing process not working as planned: as afetr [after] the first gel insertion, the baby went bradycardic for over 5 mins this then put me off having the next 2 gels as planned. I felt 1 particular senior midwife felt that I was been difficult not wanting the gels and treat me inappropr [inappropriately]  Midwives repeatedly telling me to wait for pain relief and wait even further for an examination due to being busy on rounds. | I received amazing care, I felt my dignity was guarded, and felt in safe hands at all times.  The team were brilliant and were so reassuring I felt we were both in very capable and professional hands. All in all we had a wonderful delivery - thankyou! | I was left to labour in the assessment room on my own with no midwife. The baby's head came out before anyone came to me. I had no midwifery care in labour. Precipitate labour (45 mins). Husband also felt traumatised by this experience.  Midwife should listen to patients more. |
| **Capacity for Autonomy Over Care Experiences** (CEQ ‘Own Capacity’ domain)  N=55 responders | Positive (N=2) | Negative (N=24) | Positive (N=5) | Negative (N=24) |
|  | I had an epidural and required on [an] episiotomy, both were discussed with me prior to these, and risks and benefits explained overall a very positive experience. | Induction took a lot longer than I expected given it was my second child. This made me lose confidence at times though the midwives helped keep me going  I felt out of control due to the additional pain and how things needed to escalate to the C-section which was required. | I felt completely in control until circumstances in labour meant an emergency c-section was indicated. All the staff respected my wishes and accommodated them wherever possible. | unhappy with care in labour. Midwives kept sending me home telling me I was not in labour. Would not give me any pain relief until I was 4cm. I was in too much pain in my back.  I feel I could have received an epidural from the moment I asked for one - I had to wait hours to get one. My experience in X ward was horrible but once I got into the labour suite and got the epidural I had excellent service from the midwives and they treated me so well. |
| **Conceptualising Safety**  (CEQ ‘Perceived safety’ domain)  N=68 responders | Positive (N=25) | Negative (N=10) | Positive (N=23) | Negative (N=10) |
|  | Our birth experience changed very quickly but once we were heading to theatre, despite the frantic nature, I felt safe and secure. Initially on the IOL ward the midwife seemed quite stretched and was very helpful when she was with you, but some hard to get hold of.  I cannot thank the midwives enough for helping me through labour and making sure I was ok throughout it. My blood pressure was well kept controlled thanks to the midwives. | I would have liked to be examined how dilated I was when my contractions got closer. As someone who had a 3A (third degree) tear I felt I was being told by the midwives to push a bit too soon and I told them no I am not pushing, I actually held back pushing as I was so scared to tear.  I felt frightened and thought something was wrong even though I was told it was okay. I felt neglected as I was not given Gas & Air when I asked for it. induction was not as I imagined. I was terrified during the induction. | It was quite a traumatic experience for me, but I felt well informed and felt in very safe hands all the way through  I cant thank my midwife and the whole team at the X for the safe delivery of my baby boy. X my midwife was incredible and i felt so safe with her during such a scary time.  Although ended up not as expected all care given was very supportive and I felt everyone was in control and made everything feel calm. | conflicting information about dilatation in labour made for a more traumatic birth experience.  It seemed that the diagnosis of pre-eclampsia was only made at the point when they did the caesarean [Caesarean]. My organs were swollen and the doctor said she wouldn't have delivered normally. |
| **Lack of Shared Decision-Making**  (CEQ ‘Participation’ domain)  N= 12 responders | Positive (N=0) | Negative (N=5) | Positive (N=0) | Negative (N=7) |
|  | - | This was a different labour for me: the drip & monitor made it very uncomfortable and I would have liked to be able to move around. | - | Found it very hard to answer some questions due to labour happening very quickly, overall found it quite intense. was all quite a lot to take in after being told it was potentially false labour as this was my first child. Due to guidance as well was no time to discuss / change position or have any pain relief. |
| **Other Experiences of Labour and Birth**  N= 64 responders | Positive (N=12) | Negative (N=16) | Positive (N=16) | Negative (N=20) |
|  | Delighted in every way with the way things happened.  Although my birth didn't go to plan (ended with EMCS) I was happy with the care I received and could not be happier with the experience. | Felt that experience with delivering the placenta negatively impacted birth experience.  I was not induced in the end. After sweep on Friday X I was 2cm dilated. The ANW did not want to use other methods to speed up labour. Labour ward was extremely full. 2 days later my waters broke and I went to full contractions every minute. There was no time for pain relief which made the....experience very challenging, scary and negative. No time for options. | Overall a great experience.  All in all we had a wonderful delivery - thankyou! | It wasn't a good experience, the whole pregnancy wasn't a good experience  Had hormone drip used this was brutal and if given the option of it I wouldn't like to have it again. Because of it I felt in too much pain all of a sudden instead of steadily. |
| **Experience of Participating in Research**  N= 19 responders | Positive (N=8) | Negative (N=3) | Positive (N=5) | Negative (N=3) |
|  | Glad I was induced when I was, happy to get earlier induction on the WILL trial.  happy that she participated because when she came in to deliver on the induction date, the team found CTG concerns if it was not for the study she may not have had a safe delivery of her baby | I feel the whole process was a shambles I came in on Thursday X, did not have my baby until X. please do not induce women at 38 weeks with hypertension. There is nothing to gain, I will never have another child at the X due to this experience. all I have taken away as a participant is a heap load of physical mental and unnecessary stress. please do not induce babies that aren't ready.  Induction date was Wednesday X. They coudn't get me a bed on the labour ward until Monday X. I was made to stay in the hospital for 5 days waiting for a bed. Really bad experience. Would not recommend, as induction date is not guaranteed. | Happy to have participated in research study  This woman had a very positive child birth experience compared to her last birth. Was happy participating in the WILL study | I really felt my induction should have been much earlier due to my Baby being predicted as big. But even at a scheduled 39 weeks induction I did not have my baby until 39+6 because of how busy the hospital was.  I strongly believe a way should be made possible for women who agree to take part in this trial so they do not wait as long as I did. I felt frustrated and anxious at some point because of the many hours of waiting for a bed to break my waters to start my induction. |

*CEQ (Childbirth Experience Questionnaire), wks (weeks)*

** Categories are not mutually exclusive.*

*† N=13 comments were not included in the analysis, as they were uninformative: "Everything was Fine"; “Some questions difficult to answer in view of elective LSCS ('I felt I could have a say whether I could get up and about or lie down', 'I felt I could have a say in deciding my birth position')”; “Thank you”; “No comment”; “Nothing else to share”; “No”; “No further comments to add”; “N/A”; “I had an elective section so some of these questions are not applicable”; “no additional comments”; “scores are based on caesarean section delivery”; “nothing to add”; “This lady had elective CS and did not feel able to answer the questions with missing answers”.*

**Figure S1**: Timing of birth for all women randomised in WILL, according to pre-pandemic and pandemic epochs


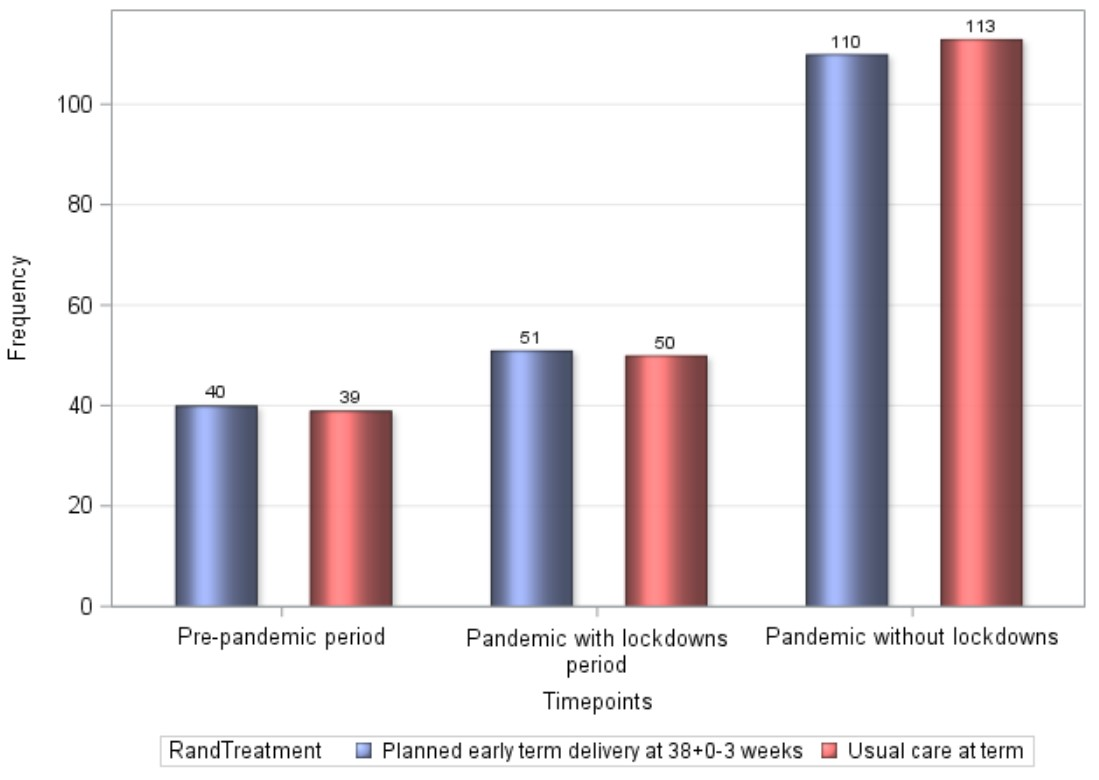

Supplement: Supplementary file 1 — Figure S1. Timing of birth for all women randomised in WILL, according to pre‐pandemic and pandemic epochs. Table S1. The WILL Trial Study Group. Table S2. Childbirth Experience Questionnaire 1. Table S3. Directed content analysis of free‐text comments by Childbirth Experience Questionnaire responders* (N, % responders). [file BJO-132-1426-s001.docx]
